# Supplementary material for: Loss of allosteric regulation in α-isopropylmalate synthase identified as an antimicrobial resistance mechanism
Source: NPJ Antimicrob Resist. 2023 Jul 3;1:7. doi: 10.1038/s44259-023-00005-4 (PMC11057210; doi:10.1038/s44259-023-00005-4)
Supplement: Supplementary file 1 — Supplemental Material [file 44259_2023_5_MOESM1_ESM.pdf]

Supporting Information for

Loss of allosteric regulation in α-isopropylmalate synthase identified as an antimicrobial resistance mechanism

Jaryd R. Sullivan[1,2,3], Christophe Courtine[1,6], Lorne Taylor[4], Ori Solomon[1,2,3], Marcel A. Behr[1,2,3,4]

[1]Infectious Diseases and Immunity in Global Health Program, Research Institute of the McGill University Health Centre, Montreal, Canada.

[2]Department of Microbiology & Immunology, McGill University, Montreal, Canada.

[3]McGill International TB Centre, Montreal, Canada.

[4]Clinical Proteomics Platform, Research Institute of the McGill University Health Centre, Montreal, Canada.

[5]Department of Medicine, McGill University Health Centre, Montreal, Canada

[6]Present address: Department of Microbiology and Immunology, Geisel School of Medicine at Dartmouth College, Hanover, NH 03755.

Marcel A. Behr
Email: marcel.behr@mcgill.ca

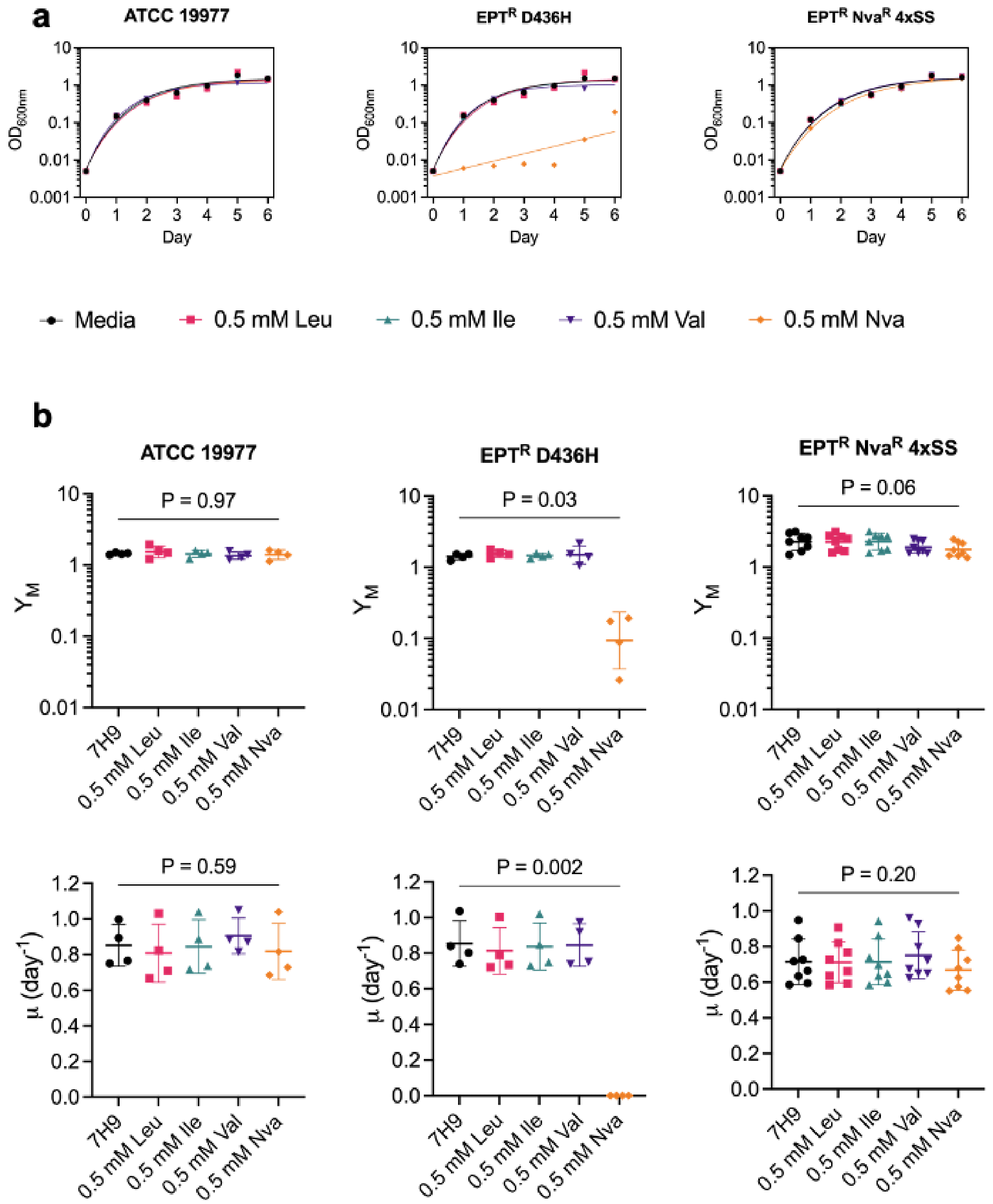

Supplementary Figure 1. Growth characteristics of *M. abscessus* EPT^R Nva^R mutant in 7H9 media. **a** Growth curves of *M. abscessus* ATCC 19977, *M. abscessus* EPT^R D436H, and a mutant with dual EPT^R and Nva^R resistance raised at 4×MIC_{90} SS. Strains were grown statically in 96-well plates with nutrient rich 7H9 media ± BCAAs and fit with exponential plateau regression. Data is representative of n = 4 independent experiments with mean ± SD of technical triplicates. **b** Comparison of growth rates (**μ**) and maximum growth plateau (Y_M) in 7H9 media ± BCAAs. Data is mean ± SD from n = 4-8 independent replicates. P values were obtained by one-way ANOVA with Dunnett's multiple comparisons test.

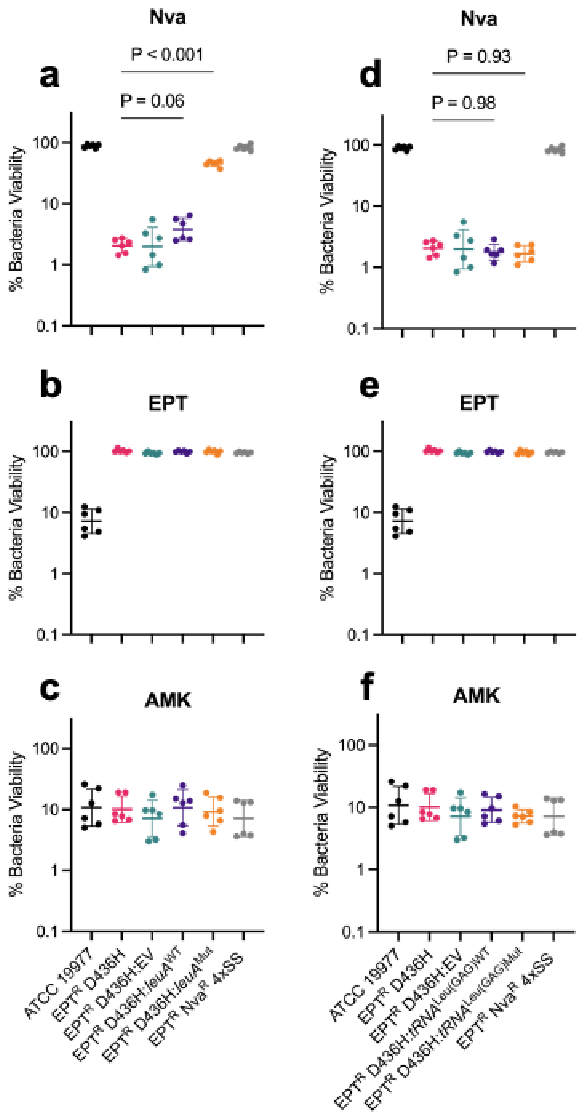

Supplementary Figure 2. *M. abscessus* EPT^R D436H strain complemented with putative *leuA* or *tRNA*$^{Leu(GAG)}$ resistance variants. Reference strain ATCC 19977 (black), EPT^R D436H (pink), EPT^R D436H complemented with empty pMV306 vector (green), complemented with wild type *leuA* (purple) or complemented with mutant *leuA* (orange), and naturally raised EPT^R Nva^R 4×SS mutant (grey) grown in **a** L-norvaline (0.6 mM), **b** epetraborole (0.3 µM), or **c** amikacin (12 µM). Alternatively, EPT^R D436H was complemented with wild type *tRNA*^{leu} (purple) or mutant *tRNA*^{leu} (orange) and grown in **d** L-norvaline (0.6 mM), **e** epetraborole (0.3 µM), **f** amikacin (12 µM). Data is n = 2 independent experiments with mean ± SD of technical triplicates. P values were obtained by one-way ANOVA with Tukey's multiple comparisons test.

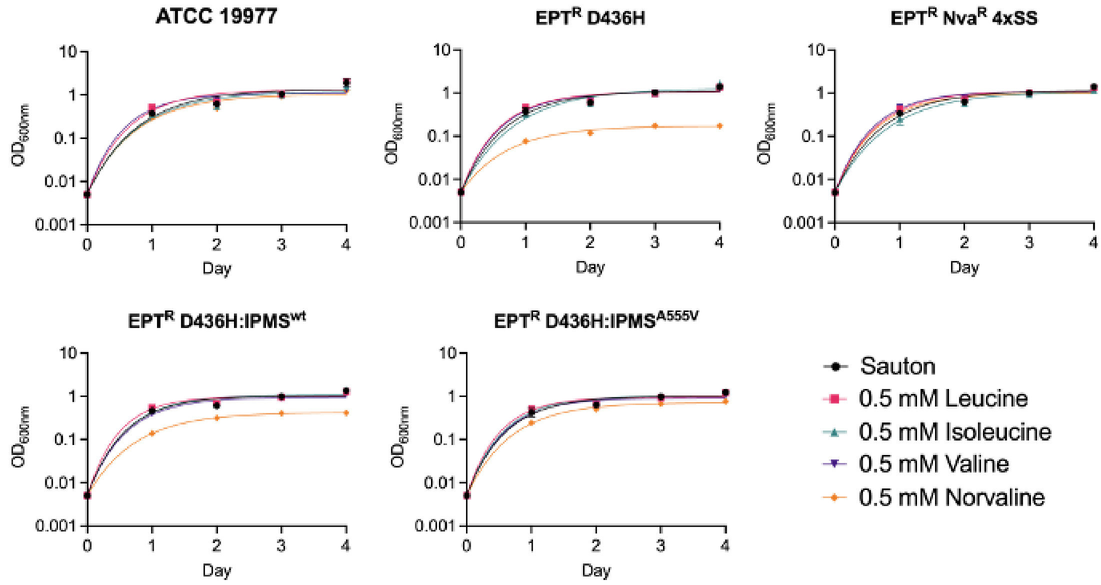

Supplementary Figure 3. Growth curves of *M. abscessus* EPT^R D436H complemented with *leuA.* Reference strain ATCC 19977, EPT^R D436H, EPT^R D436H complemented with wild type *leuA* or mutant *leuA,* and naturally raised EPT^R Nva^R 4×SS mutant were grown statically in Sauton's minimal media ± L-leucine (pink squares), L-isoleucine (green triangles), L-valine (purple inverted triangles), or L-norvaline (orange diamonds) at 0.5 mM and fit with exponential plateau regression. Data is representative of n = 3 independent experiments with mean ± SD of technical triplicates.

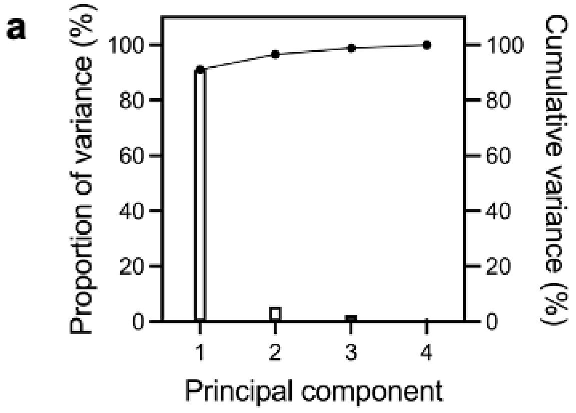

b

Strain	GroEL (MAB_0650) Counts[a]	FC[b]	P-value[c]	Acyl-CoA deH$_2$ase (MAB_4437) Counts[a]	FC[b]	P-value[c]
ATCC 19977	584	1	n.d	40	1	n.d
EPT^R D436H	2943	5	<0.0001	277	6.9	<0.0001
IPMS^{WT}	3002	5.1	<0.0001	135	3.4	<0.0001
IPMS^{A555V}	1123	1.9	<0.0001	28	0.7	0.19
EPT^R Nva^R 4xSS	1695	2.9	<0.0001	40	1	0.25

[a]Quantitative spectral counts; [b]Fold change relative to ATCC 19977; [c]P-value from Fisher's exact test with Benjamini-Hochberg correction; n.d, not determined.

Supplementary Figure 4. Proteomic analysis of *M. abscessus*. Cell lysates were extracted from *M. abscessus* ATCC 19977, *M. abscessus* EPT^R D436H, *M. abscessus* EPT^R Nva^R 4×SS, *M. abscessus* EPT^R D436H complemented with IPMS^{WT} or IPMS^{A555V} grown in Sauton's media with 0.5 mM L-norvaline for 24 hours. **a** Variance associated with each principal component. PC1 accounted for 90% of the variance, PC2 accounted for 5% of the variance. **b** Quantification of GroEL (largest PC1 coefficient) and Acyl-CoA dehydrogenase (largest PC2 coefficient) abundance.

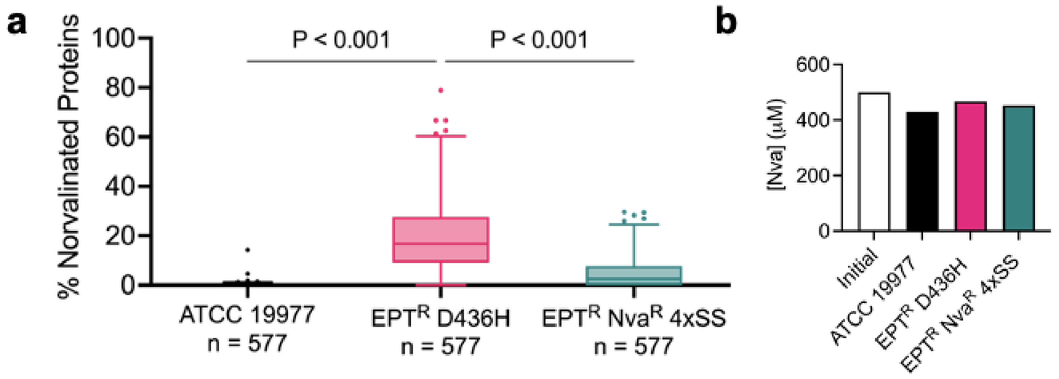

Supplementary Figure 5. Norvalination of the *M. abscessus* proteome. **a** Percentage of proteins with misincorporation of L-norvaline at leucine residues from cell lysates after 24 h of growth in Sauton's media with 0.5 mM L-norvaline. Data is median with IQR, whiskers represent 1-99 percentile. P values were obtained by Friedman test with Dunn's multiple comparisons test. **b** Concentration of L-norvaline remaining in culture supernatants after 24 h L-norvaline challenge from **a**.

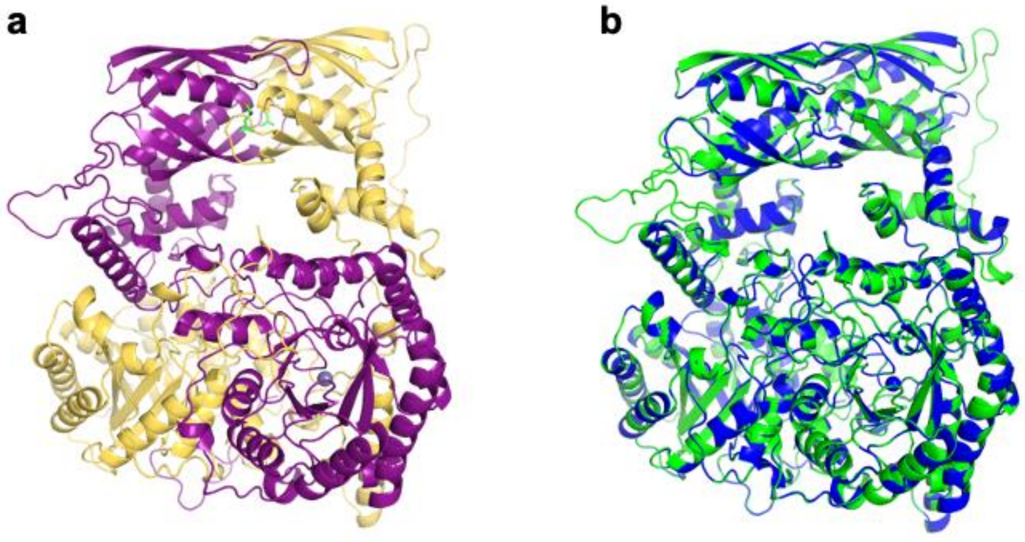

Supplementary Figure 6. Predicting homology between **α**-IPMS$_{Mtb}$ and **α**-IPMS$_{Mabs}$. **a** **α**-IPMS$_{Mtb}$ model was generated using Swiss-Model and the experimentally determined structure PDB 3FIG with QMEAN global 0.87 $\pm$ 0.05 [1–3]. **b** Overlay of **α**-IPMS$_{Mtb}$ (green) and Swiss-Model generated **α**-IPMS$_{Mabs}$ (blue) from PDB 3FIG with QMEAN global of 0.86 $\pm$ 0.05. Overlay generated rmsd of 0.199 Å.

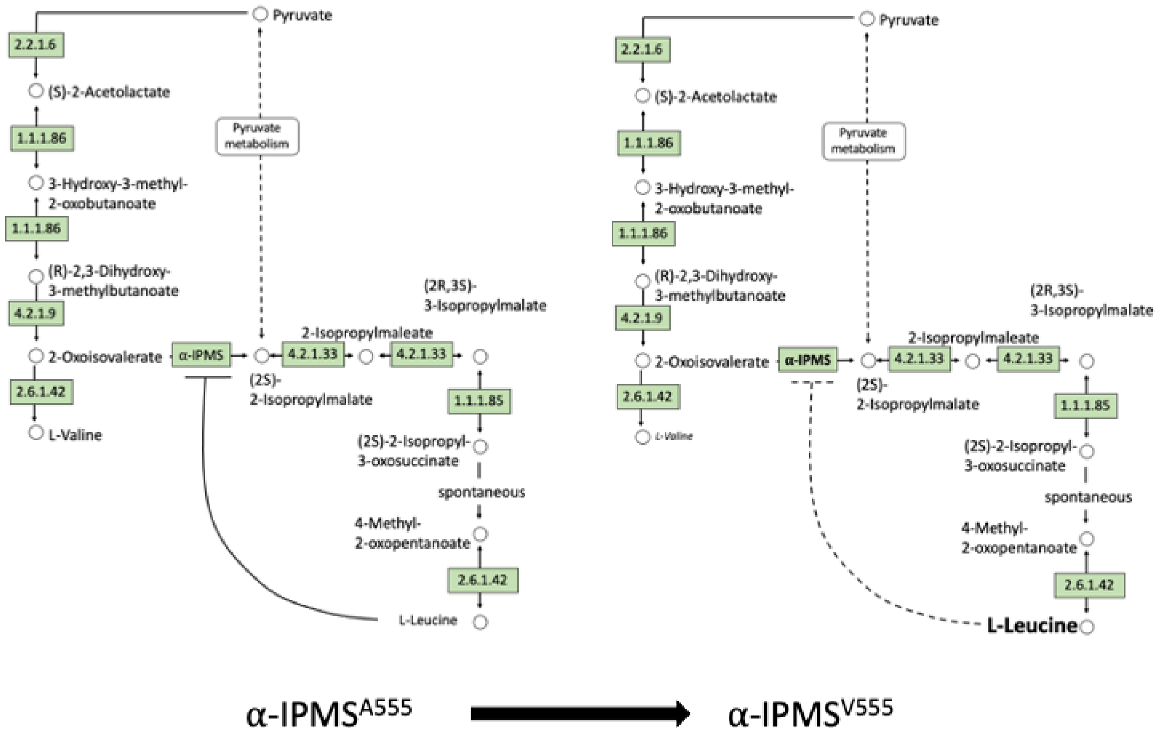

Supplementary Figure 7. Effect of **α**-IPMS mutation on BCAA metabolism. Pyruvate is metabolised into L-leucine and L-valine through a shared pathway. (Left) A negative feedback loop from L-leucine binding in the allosteric site on **α**-IPMS is maintained to ensure controlled L-leucine production. (Right) Loss of allosteric activity via a mutation in the binding site could lead to abnormal L-leucine production and impaired L-valine production through depletion of the shared intermediate 2-oxoisovalerate.

SI References

1. Koon, N., Squire, C. J. & Baker, E. N. *Crystal Structure of Leucine-bound LeuA from Mycobacterium tuberculosis*. (2008) doi:10.2210/pdb3fig/pdb.
2. Waterhouse, A. et al. SWISS-MODEL: Homology modelling of protein structures and complexes. *Nucleic Acids Res.* **46**, W296–W303 (2018).
3. Studer, G. et al. QMEANDisCo—distance constraints applied on model quality estimation. *Bioinformatics* **36**, 1765–1771 (2020).
